# Interactive Cumulative Burden Assessment: Engaging Stakeholders in an Adaptive, Participatory and Transdisciplinary Approach

**DOI:** 10.3390/ijerph15020260

**Published:** 2018-02-03

**Authors:** Rehana Shrestha, Johannes Flacke, Javier Martinez, Martin van Maarseveen

**Affiliations:** Faculty of Geo-information Science and Earth Observation (ITC), University of Twente, PO Box 217, 7500 AE Enschede, The Netherlands; j.flacke@utwente.nl (J.F.); j.martinez@utwente.nl (J.M.); m.f.a.m.vanmaarseveen@utwente.nl (M.v.M.)

**Keywords:** cumulative burden assessment, environmental health, stakeholder engagement, social learning, knowledge co-production

## Abstract

Cumulative burden assessment (CuBA) has the potential to inform planning and decision-making on health disparities related to multiple environmental burdens. However, scholars have raised concerns about the social complexity to be dealt with while conducting CuBA, suggesting that it should be addressed in an adaptive, participatory and transdisciplinary (APT) approach. APT calls for deliberation among stakeholders by engaging them in a process of social learning and knowledge co-production. We propose an interactive stakeholder-based approach that facilitates a science-based stakeholder dialogue as an interface for combining different knowledge domains and engendering social learning in CuBA processes. Our approach allows participants to interact with each other using a flexible and auditable CuBA model implemented within a shared workspace. In two workshops we explored the usefulness and practicality of the approach. Results show that stakeholders were enabled to deliberate on cumulative burdens collaboratively, to learn about the technical uncertainties and social challenges associated with CuBA, and to co-produce knowledge in a realm of both technical and societal challenges. The paper identifies potential benefits relevant for responding to social complexity in the CuBA and further recommends exploration of how our approach can enable or constraint social learning and knowledge co-production in CuBA processes under various institutional, social and political contexts.

## 1. Introduction

Environmental health issues are receiving attention in many international initiatives such as the Ottawa Charter [1] and the Health in all Policies discourse [2,3]. Environmental legislation and regulations for individual environmental pollutants such as the Clean Air Act [4] for air quality or Noise Action Plans [5] for noise nuisance have attracted considerable attention in Europe, particularly in relation to reducing pollution levels. However, scholars have pointed out the need to identify and address health disparities with regard to multiple environmental burdens and benefits across population having varying social vulnerability [6]. Framing the disproportional distribution of environmental burdens and resources as an issue of distributional environmental justice [7], both academic scholarship and public policy initiatives have advocated use of cumulative burden assessment (CuBA) to inform planning and decision making [8,9,10,11].

A full spectrum of CuBA may include the complex dynamics of compounding, aggregating and interaction of various benefits/burdens This includes driving forces such as urban development, zoning decisions, and economic and social processes that put pressure on the environment, such as increases in traffic density, emissions and waste release [12]. Both driving forces and pressures contribute to the state whereby the environment is often degraded through increases in air pollution and noise nuisance, facilitating population exposures that might produce health disparities among populations of varying social vulnerability. However, CuBA, as the term used in this study, indicates the simultaneous occurrence of a number of environmental features at the same time and in the same place. It integrates a range of environmental burden and benefits that represent the state of the environment (e.g., air pollution, noise pollution, green areas, forest areas) with a range of social vulnerability factors, such as the socio-economic status of the population, into one or more indices. Such an indicator-based CuBA can be used to identify populations and places that are subjected to both elevated environmental exposures and lack economic, political and social resources for either avoiding or adapting to the actual environmental situation. In contrast to a health impact assessment, which quantifies potential health impacts of a given plan or development project [13], a CuBA serves as a screening tool to identify ‘hotspots’ that require additional study, investments, or other precautionary actions [9,10,14]. Therefore, conducting a CuBA offers opportunities for addressing environmental health related socio-spatial inequalities in planning and policy processes.

Various indicator-based approaches to CuBA are being developed at regional to local scales [15,16,17,18]. The shift from burden assessment of single environmental factors to assessment of cumulative burdens is considered important for understanding the socio-spatial characteristics of health inequalities [16]. Nonetheless, it has brought new challenges that are both technical and social in nature [14]. The technical challenges comprise limited data availability for various factors, uncertainty of interaction among various environmental factors, and uncertainty regarding combined health effects of multiple environmental stressors [8,19]. In particular, concerns have been raised about dealing with ‘social complexity’ while conducting CuBA. As Huang and London [14] have noted, the ‘social complexity’ associated with CuBA shares the basic characteristics of a ‘wicked problem’ [20]. Similarly to a ‘wicked problem’, a clear problem definition in CuBA is controversial as it is driven by a number of individual, institutional, behavioural and structural factors. Identifying and analyzing significant factors in CuBA amidst multiple perspectives of stakeholders do not follow linear, easily-standardized or agreed formulas. CuBA could not, therefore, be supported only by providing better data and expert-based assessment ([21] in [14]). CuBA needs to be place-specific to consider unique characteristics and at the same time consistent with accepted standards of scientific rigor and regulatory frameworks [14]. Although the knowledge of multiple stakeholders from both science and practice is required to address cumulative burdens, multi-stakeholder knowledge and interest may remain contested and the approach to address cumulative burdens can have inherently unintended consequences.

Several researchers have called for an adaptive, participatory and transdisciplinary approach (APT) to address such ‘wicked problems’ [22,23]. Huang and London [14] argue that if CuBA is conducted in an APT manner it may address some of the ‘wickedness’. In particular, APT calls for deliberation among various stakeholders so that rather than trying to ‘solve the wicked problem through standard science-based approaches, these problems are tackled, managed and dealt with’ by sharing knowledge and engaging in a social learning process [22,23]. In this regard, enabling an active dialogue, encouraging a culture of social learning and the explicit co-production of knowledge by linking ‘domains of discourse’ of various stakeholders are pivotal to APT. To link ‘domains of discourse’—i.e., contexts for a reasonably coherent exchange of arguments and ways of knowing [24]—among various actors with their specialized knowledge requires a science-based stakeholder dialogue as an interface for the combination of different knowledge domains. There is a long-standing tradition of facilitating stakeholder dialogues in decision-making processes regarding complex environmental problems [25,26]. However, the practice of a science-based stakeholder dialogue—unlike ‘public engagement’, ‘policy dialogues’, and ‘multi-stakeholder dialogues for governance’—is seen as a structured communicative process for linking different types of knowledge [27].

To induce a science-based stakeholder dialogue in CuBA with the aim of enabling social learning and knowledge co-production, the approach must communicate information in the right way and enable deliberation that incorporates exchange of arguments, values and knowledge within the realm of both social and technical challenges related to CuBA. The approach needs to avoid merely producing optimal technical solutions and instead emphasize the establishment of shared goals and interests among a multitude of stakeholders through an open process. As such, the process of establishing indicators and assessing cumulative burdens needs to be an exercise embedded in a culture of active dialogue and social learning that allows for rapid adjustments and feedback loops in a participatory environment that focuses on explicit knowledge co-production among stakeholders. Moreover, CuBA needs to be situated at the interface between science and practice so that it can incorporate the production and the use of knowledge from both academia and practice alike [28]. This calls for an innovative stakeholder-based CuBA approach that enables an iterative, multi-faceted process and focuses explicitly on encouraging social learning and knowledge co-production.

Following this reasoning, we describe in this paper an interactive approach for facilitating science-based stakeholder dialogue in CuBA. In two workshops organized in German cities, one in Dortmund and another in Munich, we explored the usefulness and practicality of this approach for engaging stakeholders in collaborative work of CuBA. Our research questions were: (1) how can our interactive stakeholder-based approach support science-based stakeholder dialogue for cumulative burden assessment? (2) what insights on stakeholder-based cumulative burden assessment can be obtained from the implementation of our approach in Dortmund and Munich?

## 2. A Framework for Supporting Social Learning and Knowledge Co-Production in Cumulative Burden Assessment

Social learning can be described as learning in and with social groups through interaction [29]. Participants, when engaging in deliberation within a collaborative dialogue, learn as individuals and as a group about their problems, their goals, the perspectives of other participants and their shared context [30]. In doing so, they are engaged in cooperative endeavours of knowledge production in which knowledge is produced through interaction and mutual learning among people with different perspectives and backgrounds [31].

**Figure 1:**
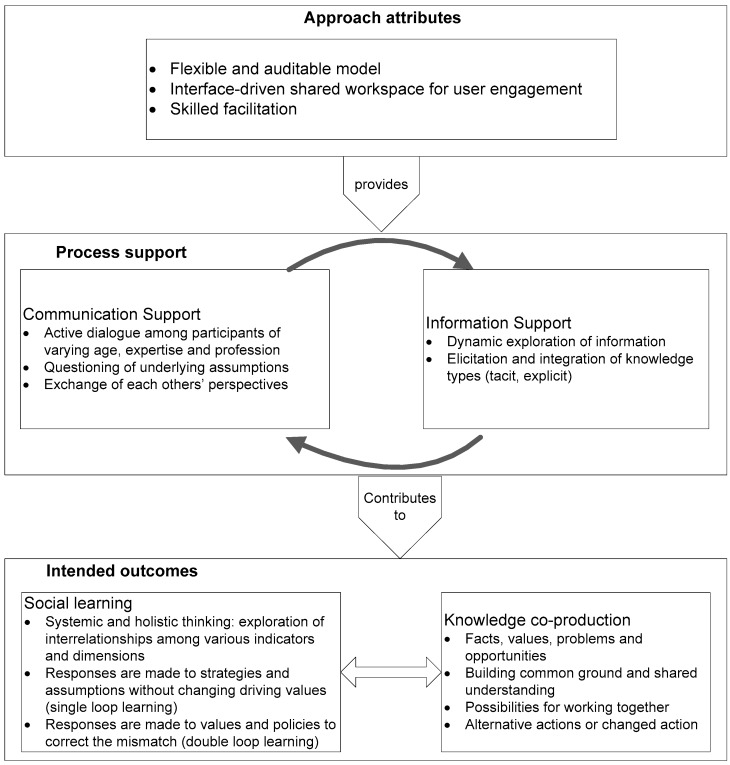
A conceptual framework for supporting social learning and knowledge co-production in cumulative burden assessment.

In addition to political, institutional and social contexts, the nature and structure of the participatory process—i.e., the participatory methods used to engage stakeholders and the processes and techniques to help stakeholders understand their own and others’ implicit frames—is equally important for fostering social learning [32]. Figure 1 outlines the specific attributes necessary for our interactive stakeholder-based approach that supports knowledge co-production and social learning in cumulative burden assessment. Our assumption is that by providing two types of process support—communication support and information support—our approach will contribute to the intended outcomes of knowledge co-production and social learning among stakeholders in relation to various issues on cumulative burdens amidst their ‘social complexity’. We synthesized the necessary attributes of our approach from recent studies that have used interactive tools during the participatory workshops. The process-support characteristics and intended outcomes were also synthesized from studies on social learning and knowledge co-production during participatory activities. Process-support and intended outcomes were later used to gather evidence for the evaluation of our approach.

### 2.1. Approach Attributes

Numerous references can be found in the literature to interactive tools that have been designed under the notion of Planning Support Systems (PSS) for supporting the informative, communicative, analytical side of spatial planning ([33], p.79). These tools have been applied in a variety of research initiatives—urban planning [34,35], environmental health [36] and renewable energy [37]—with a focus on strengthening collaboration among and between domain experts and stakeholders, as well as citizens in general. Drawing on existing PSS tools, three main attributes are considered to be appropriate when designing an interactive stakeholder-based CuBA: a flexible and auditable model; an interactive interface-driven shared workspace; and skilled facilitation. A flexible and auditable model allows stakeholders to freely select indicators or change assumptions relevant for their CuBA. It has been generally argued that participants may not use the indicators and indices the way assumed by the scientist as in many cases the topics are characterized by different legitimate interpretations of the same indicators/index [38,39]. Therefore, in CuBA process by explicitly giving users an opportunity to work with a flexible and auditable model, this may enable them to perform calculations in a way that is transparent, relevant and easy for them to comprehend. In doing so, the participants may also come to understand how assessment results are connected to choices they have made during the process, as well as enable them to explore relevant topics in varying degrees of detail or depth [40].

The process of deliberating through alternative modes of interaction—in contrast to the usual one-way interaction between science and practice—is considered essential for social learning [41]. For such alternative modes of interaction the unidirectional flow of information from the ‘research sphere’ to the ‘policy sphere’ needs to evolve into a two-way flow in which knowledge and information from both science and practice is contested, co-produced and reflected upon [42]. This is particularly relevant in stakeholder-based CuBA, in which stakeholders’ views on cumulative environmental burdens and their effect on health across varying levels of social vulnerability are determined as much by their values, knowledge and beliefs as by their knowledge of quantitative information. As such, an interface-driven shared workspace allows interactive run-times with the model, thereby facilitating user engagement [40]. This implies providing a ‘dialogue space’ where stakeholders can interact with the model by providing input or changing assumptions in the model and receiving feedback in real-time through the same interface; that feedback can be deliberated and reflected upon, and so on.

Additionally, user engagement in participatory processes requires skilled facilitation. In any participatory process, the role of facilitation is to maximize the goals of the participatory activity. A facilitator ensures that all participants feel comfortable in sharing their ideas, views and perspectives within a group. Further, facilitators display empathy with participants and provide critical feedback while simultaneously ensure that the participants remain focus on the objective at hand, as well as analyze issues that arise in depth [43].

### 2.2. Process-Support

In order to achieve the goals of social learning and co-production of knowledge, our interactive stakeholder-based approach needs to provide two kinds of support during the assessment process: communication support and information support. The communication support aims at supporting elements that are important for engendering social learning and co-production of knowledge in CuBA: active dialogue, questioning of underlying assumptions, and an exchange of each other’s perspectives. An active dialogue, or what Tsoukas [44] calls a ‘productive dialogue’, is crucial for engagement to occur among people of varying age, expertise and knowledge, and among those with differing opinions. Such dialogue occurs when participants feel comfortable in contributing their knowledge and are prepared to listen to others respectfully and with an open mind [45]. Questioning the underlying assumptions made in the model cultivates transparency in the process and supports social learning and a shared understanding of the issues under investigation [40]. By providing opportunities for participants to understand the underlying assumptions, as well for expressing their viewpoints without hampering the process, participants may be enabled to identify areas that could be focused upon constructively and to identify other areas that need further deliberation and improvement [46]. Along with explicit knowledge, insights into normative aspects and each other’s assumptions underlying the different viewpoints are needed so that stakeholders can work through discrepancies and differences among knowledge sources to produce meaningful information [47]. Therefore, participants should also be enabled to share perspectives among each other through clarification and mutual evaluation while taking part in an active dialogue.

Our approach should provide information support throughout the process by allowing dynamic exploration of information, i.e., eliciting and integrating both tacit and explicit knowledge. Participants exploring spatial information dynamically and analyzing impacts using a shared spatial language may lead to learning through improving the quality of dialogue [35]. Furthermore, Friedmann [48] argues that in order to support social learning the process needs to integrate the two kinds of knowledge—explicit knowledge and stakeholders’ tacit knowledge—to yield an understanding greater than either could have produced individually. Therefore, elicitation of stakeholders’ tacit knowledge and continuous integration of factual and empirical knowledge with stakeholders’ knowledge and any assumptions underlying the different viewpoints can enrich cumulative burden assessment.

### 2.3. Intended Outcomes

The intended outcomes to which process-support contributes are related to the characteristics of social learning process and co-production of knowledge. Various theories are available on learning that reflect on how people learn as individuals and within a group. Nonetheless, these theories have been acknowledged to be complementary rather than competitive; they provide an understanding of the processes upon which social learning is based [49,50]. For instance, learning can be instrumental according to Mezirow [51], i.e., focused on acquiring new knowledge or skills. ‘Theories of communicative learning’ describes the process by which a person constructs an inter-subjective understanding of a situation by exchanging and reinterpreting knowledge through communication with others [52]. Likewise, transformative theories of learning describe a process whereby people gradually change their views on a subject [49]. Transformative learning is analogous to ‘double-loop learning’ which is said to occur when the detected mismatch between expected and observed outcomes is corrected by revisiting the assumptions, policies and values that underlie the actions [53]. On the other hand, single-loop learning is categorized as incremental learning and is said to occur when a mismatch between expected and observed outcomes is corrected, leaving the underlying theories for the action unchanged. A feedback loop from actual experience does not change the basic assumptions or decision-making rules. Similarly, experiential learning as proposed by Kolb [54] describes a process in which concrete experiences of people lead to reflection, and the generation of new ideas, which they apply in turn through active experimentation and, therefore, learn.

In general, altered perspectives with changes in people’s perceptions or opinions represent a measure of social learning [45,55,56]. However, a large shift in opinions or belief could not be expected within one intervention, and changes may be detected where participants lack knowledge beforehand or have not yet formed opinion [56]. Such a change occurs in response to an external ‘trigger’ that leads to critical reflection and a transformation in perspective [49,57]. The extent to which the approach has supported ‘triggering’ of the change in this study was assessed by exploring evidence in the following three instances: (i) single-loop learning is encouraged when participants are enabled to change a detected mismatch by changing their strategies or the assumptions underlying those strategies; (ii) double loop learning is enabled to respond to underlying values and policies [55]; and (iii) systemic thinking is crucial for learning as individuals adjust their mental models when they understand multiple facets of an issue [45,56]. Participants begin to think systematically when they are enabled to envisage connections and feedback loops between various components. Along this line, the participants should also be enabled to explore interrelations among various dimensions of cumulative burdens.

In the course of social learning, the knowledge co-produced in the process manifests itself in three dimensions: cognitive dimensions, a moral dimension, and relational dimension [58]. Participants acquire new information, learn facts and values on the issue, identify problems and opportunities, and form a reasoned opinion that lead to cognitive enhancement [59]. Likewise, when participants are enabled to understand concerns of others and areas of agreement and disagreement, this contributes to building common ground or a shared understanding of the issues [46,60]. This in turn lays the foundations of collaborative relationships or contributes to relational dimension by offering prospects to work together and for collective or even alternative action within a participatory activity [60].

## 3. An Interactive Stakeholder-Based Cumulative Burden Assessment

Based on the approach attributes described in the previous section, our approach on interactive stakeholder-based CuBA comprises three components—a flexible and auditable model, an interface-driven shared workspace, and skilled facilitation—which will be described in the following subsections.

### 3.1. Flexible and Auditable Model

The indicator-based CuBA method adopted in our model has been discussed in detail by Shrestha et al. [18]. Firstly, the method uses a grid to represent information in a fine spatial resolution for both environmental and social vulnerability indicators and indices. Secondly, stakeholders can deliberate on the cumulative burdens at a local level, unrestricted by administrative boundaries. Using information at small scales is particularly relevant for identifying intra-urban spatial inequalities and ‘hotspots’ of cumulative burdens. Thirdly, the method uses a relative procedure for normalization/standardization of indicators by using environmental standards and city-wide averages. In doing so, the method supports the stakeholder to understand the assessment result in a relative manner that is easy to comprehend, understand and communicate. Lastly, the method allows the integration of environmental burdens and benefits together with social vulnerability using a simple aggregation method.

**Figure 2:**
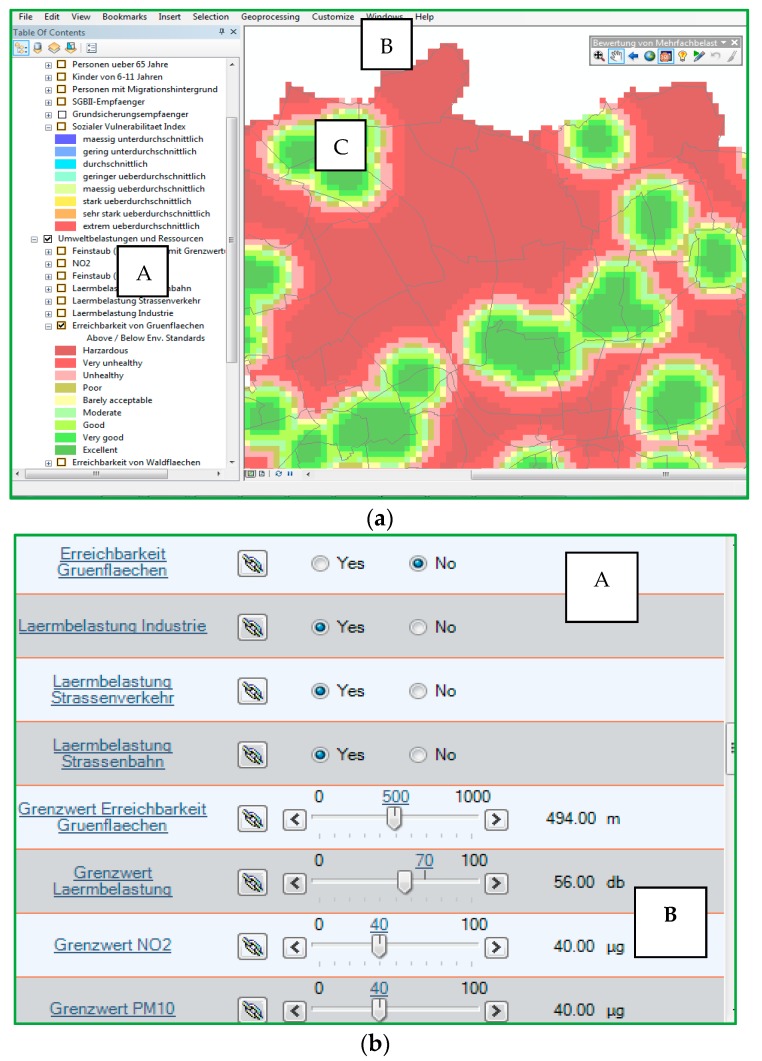
(**a**) User interface for A-overlaying indicators/index, B-manoeuvring on the maps, C-dynamic visualization of 2D maps; (**b**) Interactive tools for A-selecting indicators to construct index, B-changing thresholds.

Community Viz Scenario 360 [61], a GIS-based PSS, is used to adapt the CuBA method into a flexible and auditable model that features interactive tools and dynamic visualization. Using the interactive tool, stakeholders can choose to overlap individual social vulnerability indicators on various environmental indicators, create either a separate indices for environmental burdens and social vulnerability, or an integrated index that combines an index/indicator of environmental burdens with a social vulnerability index/indicator (Figure 2a). The tool also allows the stakeholders to select/deselect the indicators to be combined in the index and this functionality is available for constructing both environmental burdens indices and social vulnerability indices (Figure 2b). For environmental burden indicators, stakeholders can change the threshold value of environmental standards (Figure 2b). The effect of these changes and their variation with respect to the distribution of ‘hotspots’ can then be viewed dynamically in real-time and spatially in the form of 2D spatial maps (Figure 2a). 

**Figure 3:**
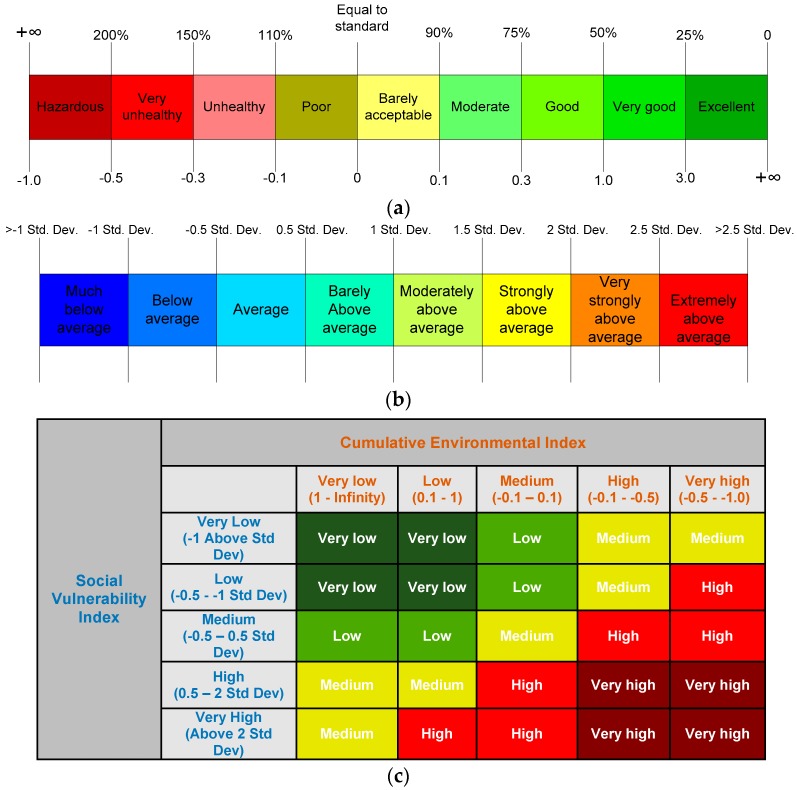
Relative scale representation for (**a**) an environmental indicator/index; (**b**) a social vulnerability indicator/index; and (**c**) an integrated environment and social vulnerability index.

To characterize and visualize the area being exposed to various levels of environmental burdens and social vulnerability the tool uses relative scale visualization. Such visualization is derived by comparing the indicator value with a set of standards or a city-wide average value and then represented by a qualitative colour-coded legend. For instance, Figure 3a shows a relative scale visualization to characterize an area with environmental burdens as developed by Vlachokostas [17]. In this scale, an approximate zero-indicator value characterizes a poor to barely acceptable exposure, which means the area has an indicator value approximately the same as the threshold value of the environmental standard. Negative values characterize a problematic situation, and the higher the environmental burden in the area the closer to −1 the indicator value will be. An indicator value greater than 1 can be considered as a very good as the value is 50% below the threshold value set by the environmental standard. Likewise, Figure 3b shows a vulnerability scale that uses the standard-deviation classification method to characterize the area compared to the city-wide average. An indicator value between 0.5 to −0.5 of the standard deviation characterizes a vulnerability score close to the city-wide average. More negative or positive values on the scale characterize lower or higher vulnerability scores, respectively, as compared to the city-wide average. In a similar manner, Figure 3c shows a qualitative scale visualization of a range of integrated environmental and social vulnerability indices. Using a matrix of environmental indices and social vulnerability indices the scale allows user to visualize the area having both environmental burdens and social vulnerability.

### 3.2. Interface-Driven Shared Workspace for User Engagement

**Figure 4:**
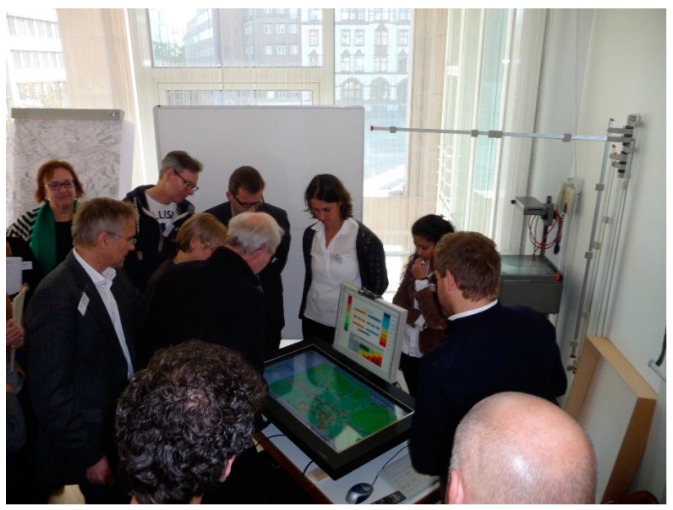
The MapTable as a shared workspace for supporting group work.

The advancement of technological development has opened up new ways for supporting a collaborative dialogue among stakeholders. Advanced hardware solutions, such as a touch-enabled horizontal screen, have made the user interface of the model more interactive and suitable for supporting group work (Figure 4).

Our approach uses a similar sort of hardware, referred to as the MapTable: a large-scale horizontal table surface with a touch-sensitive screen that shows the content as a common map interface and supports user interaction with the map via touching and gestures.

### 3.3. Skilled Facilitation

Hirokawa and Gouran [62] divide activities of facilitators into three areas: substantive, i.e., related to topics discussed; procedural, i.e., drawing attention to process elements such as the agenda; and relational, i.e., attending to social or emotional issues. Additionally, the use of models in participatory activity requires tool-related facilitation. Niederman et al. [63] argue that knowledge of a tool is a critical factor in such meetings, pointing out that the use of technology could be a barrier due to participants’ anxiety about the technology, distractions caused by the technology and the learning time needed for participants to use it. Therefore, the facilitator (referred to as the chauffeur) in our approach also supports the participants on technical issues while using the MapTable and other interactive tools.

## 4. Implementation of the Interactive Stakeholder-Based CuBA in Dortmund and Munich

### 4.1. Context and Case-Study Areas

The Jufo-Salus junior research project, entitled as ‘Cities as a healthy place to live, regardless of social inequality’ [64], was started in 2013 with the aim of integrating the concepts of sustainability and health for planning in urban areas. To achieve this the project focuses on addressing health inequalities in the urban area by highlighting both pathogenic (environmental burden) and salutogenic (environmental benefits) factors and their unequal distribution across a population that exhibits varying social vulnerability. Two German cities, Dortmund and Munich, were chosen as case-study areas for the project. Studies have shown the existence of spatial inequalities in these cities in relation to cumulative environmental burdens and their unequal distribution across various social vulnerability factors [65,66].

One central aspect of the Jufo-Salus research group’s approach is to facilitate a transdisciplinary dialogue amongst researchers from urban planning and public health disciplines, as well as practitioners from relevant fields, with regard to environmental health. Various participatory methods and approaches have been applied by the group in workshop settings at different phases of the project, such as World Café [67] and ISUSS [36] during the problem scoping and problem understanding phases. In a later stage of the project, a planning game was designed as a one-day workshop in both cities. The design of the planning-game workshop was embedded in the context of Social City Programme [68], which is an assistance programme being conducted as part of federal urban development policy in Germany. The aim of the programme is to upgrade and revitalize economically and socially deprived districts, neighbourhoods and communities. To do so, the programme intends to develop interdepartmental coordination, improve linkage between the various policy areas (e.g., employment, housing development, social integration, health promotion) and find synergies for efficient project funding schemes. Therefore, one of the tasks in the planning-game workshop was to identify the so-called programme areas, in which all stakeholders decide collaboratively to invest in during a workshop session (herein refers to one particular session where we implemented our approach during the planning game workshop). In doing so, the participants in the session were directed to consider health promotion and spatial inequalities in relation to environmental burdens as central topics and to integrate a cumulative burden assessment, mediated by our approach, as a screening tool in their discussions.

### 4.2. Participants

Intentionally, the participant selection aimed to involve both researchers and practitioners representing diverse sectors and interests in environmental health issues. In this regard, contacts from previous Jufo-Salus workshops were used to identify potential participants based on their professional roles, expertise and knowledge on environmental health issues in Dortmund and Munich, respectively. Five individuals participated in the Dortmund workshop session and seven individuals in the Munich workshop session. Participants in Dortmund included one staff member from the municipality’s urban planning department, three practitioners associated with a NGO/social entrepreneur—the local agenda21 group, a tenant association and a youth organization—and one researcher in public health. The Munich workshop session included three municipal staff members from the social planning department, environmental department and the health departments, three practitioners respectively associated with an environmental NGO, a health insurance company, and a social housing organization, and one researcher in urban planning. Two authors of this paper facilitated the workshop, the first author carried the role of a chauffeur and the second author generally facilitated the process in both workshops. Two other persons observed each workshop in its entirety and made notes of their observations.

### 4.3. Model Specification

Two separate models were prepared for Dortmund and Munich, respectively. Both models followed the general design characteristics described in Section 3. The environmental and social vulnerability indicators used in both cases are listed in the appendix to this paper (Table A1). The preparation of the model had been very selective so as to include only those environmental variables that represent external characteristics of the environment. The inclusion of indicators in the model were also influenced by the relevance of these indicators for the cities as based on previous stakeholder workshops run by Jufo-Salus and the availability of data at neighbourhood level.

The models used a 125 × 125 m grid as spatial resolution for both individual environmental indicators and the cumulative environmental index. While city-wide modelled data on air quality were acquired for this resolution, available point-data for noise nuisance in Dortmund had to be interpolated for the 125 × 125 m grid. For Munich, modelled data at the 125 × 125 m resolution were available for noise nuisance, but measured air quality data were only available along its roads. For this reason the indicators and the index of air quality for Munich were prepared as line data. Indicators on access to green areas (parks and forests) for both cities were prepared as described by Shrestha et al. [18]. In the workshops, we considered 1 ha as the minimum area required for providing recreation and opportunities for physical activity. Both social vulnerability indicators/indices and integrated socio-environment indices used a spatial resolution of 25 × 25 m, chosen after visual inspection to avoid under/over-representation of residential areas at the neighbourhood level. Social vulnerability indicators were prepared using a poly-categorical three-class dasymetric mapping of population distribution [18].

Environmental standards shown in the appendix (Table A2) were used to normalize the environmental data, whereas social vulnerability indicators were standardized using the city-wide average. Although the assessment of cumulative burdens resulting from a number of environmental factors and their impacts on health requires an understanding of complex interactions among these factors, we decided to use a simple aggregation method that is easy to comprehend by the stakeholders. Additive aggregation was used to combine individual indicators into an index for both environmental factors and social vulnerability factors. For environmental factors, additive aggregation as proposed by Vlachokostas [17] was applied, whereas simple additive aggregation as described by Shrestha et al. [18] was adopted for constructing the social vulnerability index. Multiplicative aggregation was used to combine the environmental index and the social vulnerability index in order to create an integrated socio-environment index. Both models were adopted into CommunityViz for the addition of flexibility and auditability into the model and integrated into the MapTable to provide shared-user interface (described in Section 3). As such, the participants could deliberate on the impact of environmental standards or indicators by changing the threshold values of the provided indicators or selecting which indicators to combine into index. Deliberation was, however, limited to only indicators provided in the models.

### 4.4. Workshop Session Design and Analysis

The planning game workshop in Dortmund took place on 29 October 2015 and in Munich on 7 November 2015; they were conducted in the German language. While the planning game was conducted as a one-day workshop, the workshop session on stakeholder-based cumulative burden assessment took 1.5–2 h. The workshop session began with an introduction by the facilitator on the objectives of the session followed by a description of the task on delineating programme areas. A short explanation of the CuBA model was provided, together with a demonstration of how to use the MapTable tools and hands-on experience for the participants.

Data for analyzing the workshop session were collected from four sources: screen capture of the interactive MapTable; voice recordings; observers’ notes; and a post-session questionnaire. Screen capture and the voice recording of the workshop session were carried out to capture complex interactions between the participants, between the participants and the model, and between the participants and facilitators. The recordings were later transcribed, translated into English and provided with time coding. The post-session questionnaire focused on the usefulness of the tool and on model specifications instrumental in identifying ‘hotspots’ of cumulative burdens. Participants were asked to use a five-point Likert scale to express their level of agreement with questions/statements and provide a short comment on each. The observers’ notes consisted of general observations on the participants’ interactions and their experiences while using the approach.

The analysis of the workshop sessions was based on reflexive engagement [69] of the two authors of this paper with participants during the workshop session. The use of the reflective engagement was motivated by our interest in examining each element of the framework in depth and the participants’ experience with our approach. We used exploratory analysis, especially focusing on micro-analysis of the workshop transcripts. In particular, focus on a single workshop and the use of micro-level analysis of conversations by the participants have been advocated as appropriate for understanding interactions mediated by technological artefacts, such as a model [44,70,71]. Nonetheless, we used transcripts from the two workshop sessions to broaden our empirical base. The screen captures, observers’ notes and post-session questionnaire were used to supplement and consolidate the findings from the workshop transcript.

To assess and report on communication and information support as mediated by our approach, we looked for operational treatments of each element of these two process-support in the theoretical description of the framework and the data. By simultaneously examining the theoretical descriptions and the data, the first author identified a number of activities associated with each element in the communication and process support and the evidence related to these activities. These were then re-examined by the second author independently and revised if needed. Using these identified activities to structure the evidences, we present the findings and examples of evidence that manifest these activities in Section 5. Although the elements of communication support and information support are presented separately, it is to be noted that they are interrelated and therefore influence each other. Likewise, some items of evidence exhibit the influence of more than one of these activities and elements. Evidence for social learning and co-production of knowledge was collected using the theoretical descriptions in the analytical framework. Any discrepancies discovered while collecting and analyzing the evidence were resolved through discussion between the authors.

## 5. Insights from the Workshops

### 5.1. General Observations and Participants’ Evaluation

In general, the flexible and auditable model augmented with the MapTable enabled the participants to explore the effect of individual indicators/indices in a shared workspace. Using relative scale visualization, and based on their selection of indicators, participants were able to visualize the spatial distribution of ‘hotspots’ with respect to single indicators/indices in real-time on maps. As such, the process evolved from one-way communication of information on cumulative burden assessment to a two-way process in which participants’ suggestions and knowledge were also considered during the assessment.

Nonetheless, the discussions that took place during the workshop sessions did not always flow smoothly. The use of an interactive tool and characteristics of the MapTable hardware were observed to constrain the process to some extent. We observed some anxiety and distractions related to the MapTable and the interactive tools—also reported by Niederman et al. [63]—as a limiting factor in using the tool. However, the facilitator provided guidance for participants, helping to maintain focus on the objectives and task of the workshop, as well as clarifying underlying assumptions in the model, communicating the uncertainty associated with scientific analysis, and providing participants with equal opportunities to ask questions or respond to the others’ perspectives. Likewise, the chauffeur assisted the participants to execute the moves using the tool as intended by the participants and encouraged them to embrace the dynamism. In both workshop sessions, it was observed that participants used individual indicators or overlaid single indicators on each other more often than they did for cumulative indices.

These observations also resonate with participants’ evaluations on the usefulness of the tool and model specifications for identifying ‘hotspots’ of cumulative burdens and thereby identify programme areas for investing resources from Social City (see Table 1). Overall, participants rated the use of the model augmented with the MapTable as being useful. Individual indicators were considered to be more useful than the integrated index for cumulative burden assessment. The added value of an integrated environmental and social vulnerability index was found to be limited in the Dortmund workshop, so it was not included in the Munich workshop. The majority of participants rated the social vulnerability index as more useful than the cumulative environmental index. They considered information on both environmental and social vulnerability factors to be useful in the delineation of programme areas for the funding of the Social City programme.

### 5.2. Communication Support and Information Support

To provide communication support for stakeholder-based CuBA we identified three main elements that our approach needs to facilitate: active dialogue among participants having varying levels of expertise and professional backgrounds; critical questioning of underlying assumptions incorporated in the model; and open exchange of each other’s perspectives. In both workshops it was observed that there was an active dialogue among the participants on various issues related to CuBA. Activities that encourage an active dialogue, such as openness and the freedom to share points of view [72], thus building on meanings proposed by others to produce alternative meanings [71], were evident as presented in Table 2. The MapTable was observed to support such active dialogue by providing a shared workspace in which indicator maps functioned as a common language. This corroborates the findings of Hopkins et al. [73], who found increased interaction among groups working with horizontal instead of vertical displays. The active dialogue was further supported by the facilitator, as indicated in Table 2.

Critical questioning of underlying assumptions of the model was also observed during the workshops. By providing opportunities to interact with the model, for example by changing threshold values of environmental indicators and viewing the result of the change in real-time, the participants were prompted to first understand the assumptions and methods used in preparing the indicators, indices and data used for deriving each indicator. In doing so, the participants were triggered to raise their concerns about various aspects of the model and to critically discuss the relevance of each indicator and index for the identification of ‘hotspots’ of cumulative burdens. Furthermore, they were stimulated to seek more information to allow them to fully understand the model. Moreover, they provided feedback related to the information used in the model (Table 2).As a result, underlying assumptions in the model became transparent and thus open to scrutiny. Mostly exchange of perspectives among participants centred around the indicator maps and on information that was acknowledged to be important but had not been included in the model used during the workshops. Further, the exchange of various perspectives on the same topic from different participants was also evident (Table 2). To conclude, the model in the MapTable provided supporting material to stimulate participants to talk with each other and was not just a source of information [74].

There are two main elements needed for the provision of information support in the process of stakeholder-based CuBA. These are a dynamic exploration of information, and elicitation and integration of both tacit and explicit knowledge. The findings presented in Table 2 demonstrate that dynamic exploration of spatial indicators and indices was well supported by our approach. Often participants were focused on exploring indicators on a particular segment of a street or neighbourhood, as well as comparing the area with the rest of the city. Such dynamic exploration of information at various scales was found to be supported by the interactive features of the tool, such as zooming in and out.

The flexibility of the model augmented with the MapTable and supported by the chauffeur also stimulated participants to explore the distribution of ‘hotspots’ with various combinations of indicators—e.g., individually or in multiple combinations to form an index, overlaying one indicator with another, and the effect of changing threshold values. In addition, the use of fine-scaled spatial units to derive indicators/indices further supported deliberation about cumulative burdens at local levels in order to identify programme areas requiring resources from the Social City programme other than those areas predefined using administrative boundaries. Elicitation of participants’ tacit knowledge and combining it with explicit knowledge seemed to emerge in multiple ways. While the participants were engaged in exploring the model using the MapTable, they used their tacit knowledge to either help others to understand the information being presented through the indicators/indices or even to highlight relevant information that was not captured in the data and indicators (see Table 3). Further, evidence indicated that the participants combined both explicit and tacit forms of knowledge and that in doing so they tended to contextualize meaning to information, to highlight relevant personal experience and to elicit their responses.

### 5.3. Intended Outcomes

#### 5.3.1. Social Learning

The communication and information support provided by our approach helped to ‘trigger’ social learning to a certain degree. During the workshop sessions, participants were encouraged to think systematically and holistically while assessing cumulative burdens and finding ‘hotspots’ of cumulative burdens. By offering them the possibility of using the tool in ways they think to be appropriate, participants were stimulated to think critically about each indicator/index. In doing so, they reasoned about the relevancy of each indicator/index in CuBA to identify areas with cumulative burdens and attempted to conceive the underlying interrelations among them. As a result, instead of creating a single index for both environmental and social vulnerability indicators, the participants in Dortmund overlaid the social vulnerability index with the single environmental indicator of access to green areas and noise from street to delineate programme areas there. In the Munich workshop, the participants created a vulnerability index based on indicators relating to older adults, people with a migration background and children aged between 6 and 14 years. By overlaying this onto the noise index they assessed cumulative burdens and delineated a programme area there. The current practice of undertaking cumulative burden assessments using quantitative indicators only was challenged during the workshops. For instance, the participants concluded that the tool lacks information on the quality of green areas, which cannot be derived from quantitative data alone. Input based on experiences and perceptions is needed, demonstrating that it is hard to delineate areas exclusively from quantitative indicators. Likewise, perceptions of people in the neighbourhood regarding noise pollution and their needs were also acknowledged to be necessary inputs in such an assessment.

Evident instances of single-loop learning in the workshops were mainly related to improving the existing CuBA model. For instance, the use of 70 dBA as a threshold for acceptable noise pollution was decided to be very high. By observing the distribution of 70 dBA noise levels participants confirmed that the areas they are aware of having high noise levels did not become visible with 70 dBA assumption. As a result, indicators for noise pollution were reduced to 55 dBA before continuing with the assessment in Dortmund. Similarly, the use of noise level indicators from various sources (train, road, industry) as separate indicators in the index was realized to have a compensatory effect in Dortmund. Therefore, the use of a logarithmic scale for combining noise levels from different sources was considered necessary in the CuBA model; this measure was confirmed during the Munich workshop. The use of 1 ha as a minimum size for green areas that was initially approved by the participants was later reconsidered as smaller parks in the city centre were filtered out using the 1 ha threshold. General shortcomings and difficulties in combining environmental indicators into an index were discussed extensively, in particular the loss of information using an aggregated index and the fact that single indicators having very high or low values are possibly averaged out in an index. For instance, one participant stated that certain indices are more useful than others. In the case of CuBA positive and negative environmental indicators need to be combined, i.e., one would still need to look at these indicators individually. In this respect a conceptual mismatch was detected while aggregating environmental factors. Areas showing high values with respect to one environmental indicator were assessed to be only moderately affected due to compensation by lower values of another indicator. For instance, areas with high exposure for noise levels were not identified as having high cumulative burdens if their accessibility to parks was considered to be good or if they had lower levels of air pollution. On the other hand, the social vulnerability index was found to emphasize areas that are vulnerable with regard to all individual indicators and is, therefore, aligned with how participants perceive social vulnerability in their city. A need to combine the knowledge and experience of participants from the area was also found to be necessary in CuBA. This was evident when a participant acknowledged that although they found one area where noise burden, problem of accessibility and social vulnerability overlap, there could be other areas depending on the indicators they have chosen. Nonetheless, they agreed on the area they have delineated as it was verified through their knowledge and experience. Likewise, the aggregated indices was found useful particularly as a screening tool to compare one area of a city, e.g., the Nordstadt, to other parts of the city, as acknowledged by a participant in the Dortmund workshop session.

Double-loop learning was observed in only a few instances. Instances of double-loop learning occurred when the participants discussed the procedure for conducting CuBA and the challenges associated with CuBA in the context of on-going policies. For instance, in relation noise levels, participants in Munich agreed on the importance of including the level of satisfaction of people, particularly in relation to noise pollution. It was suggested to assess noise pollution at two levels, one based on the data and another based on subjective experiences, and then to integrate these to identify areas with stronger impacts. The participants in Munich concluded that areas with cumulative burdens are not confined to a fixed administrative boundaries—meaning that different administrative units and boards are responsible—which ultimately makes it difficult to assess and plan interventions. Similarly, the issue of the weighting of each indicator—either based on its impact on health or on the objective of the assessment—was raised and at a later stage dismissed, suggesting that weighting often includes political decisions.

#### 5.3.2. Knowledge Co-Production

The presence of attributes of social learning process in the participatory activity lead to co-creation of knowledge among the groups. The co-creation of knowledge has been identified to be manifested in three aspects: (a) a cognitive dimension or acquiring knowledge about facts, values, problems and opportunities; (b) a moral dimension or building common ground; and (c) a relational dimension, such as establishing a collaborative relationship, for undertaking collective action. Cognitive enhancement was evident during workshops as participants clearly opted to acquire knowledge other than that previously held. For instance, the transport planner told the facilitator that although he is not a social expert he found the social vulnerability index useful. Another participant who had been living in Dortmund already for 40 years claimed to have knowledge about the green areas and therefore wanted to know about the impact of road traffic on these areas. In the Munich workshop one participant from the health department observed that there was much more social data available than she had known about before. Discussions also sprang up in which participants shared their perspectives on values such as ‘what is more risky: noise or PM_10_?’ Problems and opportunities for assessing cumulative burdens and thus identifying programme areas were identified jointly. For instance, the interactive properties of the model, relative scale normalization and real-time visualization were useful in supporting stakeholders in their deliberations about cumulative burdens, whereas challenges were related to aggregation of environmental indicators into indices without compensating the effects of each other.

Building common ground in participatory activities refers to identifying areas of agreement upon which to focus constructively and, similarly, areas of disagreement that need further deliberation. The participants discovered common linkages between various interests. For instance, in the process of exploring the distribution of children as an issue of interest of one participant, another participant shared an interest in knowing the distance children at various locations have to travel to visit parks. The participants therefore combined the indicator of distribution of children with that of accessibility to green areas. In this way, the participants were able to work through their interests linking these with others, so that later they were able to agree on a set of indicators/indices for assessing cumulative burdens so as to delineate programme areas. Collaborative relationships were also evident in the form of opportunities for different departments to work together. In the Munich workshop, participants remarked that the model and the MapTable could be used to integrate data from various departments, e.g., health, environment, social, education, and to overlay these to generate new, meaningful information. Finally, the approach lead to collective action by the group: new programme areas were delineated that integrated cumulative burdens and their impact on social vulnerability, as well as knowledge of multiple stakeholders.

## 6. Discussion and Conclusions

The ‘social complexity’ with respect to CuBA has generated a growing need to focus on engaging stakeholders in social learning and the knowledge co-production process through the practice of a science-based stakeholder dialogue as an interface for combining different domains of knowledge. Although social learning may occur whenever stakeholders come together to discuss their differences, the learning opportunities that arise require careful nurturing [49]. Individuals learn and knowledge is co-produced when explicit knowledge (e.g., scientific information, data) is taken up in their cycle of knowledge acquisition, so that such explicit knowledge is made sense of through their own tacit knowledge base (experiential knowledge, know-how) in an open and collaborative process [75]. In this regard, the approach presented here clearly supports such a science-based stakeholder dialogue in CuBA. The insights from the two workshops demonstrate that the approach has been able to position the CuBA process in the interface between the production and use of knowledge from both research and practical points of view. By opening a window of opportunity for interaction with a flexible, auditable model in a shared workspace provided by the MapTable and supported by a facilitator, the stakeholders were enabled to deliberate collaboratively on cumulative burdens, to learn about the technical uncertainties and social challenges associated with cumulative burdens, and to co-produce knowledge in a realm of both technical and social challenges.

A number of potential benefits from the approach can be identified that are relevant for addressing ‘social complexity’ in CuBA. Firstly, it is acknowledged that in addition to expert-based scientific reasoning and knowledge from a broad range of stakeholders, CuBA requires the incorporation of ethical considerations, multiple perspectives and value judgements. To meet such requirements our approach enabled a group of stakeholders to actively and collaboratively engage in the process of cumulative burden assessment. In doing so, stakeholders tended to exchange their perspectives and integrate their tacit knowledge, values and reasoning in the process, as was evident from the workshops. Secondly, in CuBA it is difficult, if not impossible, to identify and analyze significant factors amidst multiple perspectives as this does not follow a linear, easily-standardized and agreed upon formula. There is thus a risk of imposing simplified models and expert-based assessment over the complex realities of CuBA [14]. In our approach we addressed this risk by providing stakeholders the opportunity to critically reflect on any underlying assumptions used in the model and on the indicators/indices included in it and to decide on their relevancy in the CuBA process. Moreover, the approach stimulated the participants to use the tool as they thought appropriate for CuBA; it was not necessary to arrive at a single index. This lines up with what Payne-Sturges and Lawrence ([76], p.3) stated: ‘perhaps the assessment or evaluation of cumulative burdens do not necessarily mean we must arrive at one number’. Thirdly, a CuBA needs to reflect the specific characteristics of a specific geographic area or population, but at the same time it must conform to scientific rigour and regulatory frameworks. This calls for ‘a mutual and recursive relationship between analysis and deliberation’ [77] in the CuBA process. The approach supported an analytic-deliberative process in CuBA by reconciling both analytic components (e.g., technical data, methods, environmental standards) and the deliberation components from the users (e.g., discursive arguments, logics, reasoning, perspectives, understanding of the issues) that are socially and contextually relevant. Fourthly, the impacts of cumulative burdens often cannot be fully predicted for the areas due to interlinking between issues, making it difficult to follow one course of action. Nonetheless, the resulting assessment, enriched with the knowledge and perspectives of many, as well as inputs of shared interest and informed choice, is considered to contribute to better informed decision-making [78]. Moreover, integration of knowledge and learning, together with increased trust, ownership and support for the assessment product, are considered to be important outcomes of the participatory process [79,80]. In this regard, our approach was set up to improve assessment outcomes by supporting stakeholders in an experimental process of step-wise inquiry into cumulative burdens. In doing so, it allowed different ways of knowing by researchers and practitioners to be integrated in an open, flexible and transparent manner, thereby engendering social learning and knowledge co-production in CuBA processes. Consequently, the approach enabled access to data and knowledge on the sectoral specificities of the participants, laid a foundation for developing common linkages among various sectors, and provided a platform for stakeholders to interact in a complementary manner, thus contributing with their respective strengths to the CuBA process.

In spite of having essential values in engaging stakeholders from both research and practice in a CuBA process, it is necessary to stress challenges pertinent to our approach. Direct interactivity with models and tools demands a high level of engagement by participants. Such levels gives users more control of the model with which to follow through on ideas about what is visualized and discussed among groups, to revisit observations, and to receive and give feedback on their interpretations and choices [81]. For instance, participants could select their own set of indictors/indices and visualize these in the MapTable. It may, however, make stakeholders anxious about understanding the model structure and the groups may even get distracted by using the tool, which was evident in a few instances during the workshops. Therefore, one of the challenges in the use of our approach is to maintain a balance between the experiences of users in attempting to understand the model’s structure or applying the tools and to put these to use when deliberating on CuBA. The use of a relative scale for coherent representations of indicators/indices and a colour coded legend was found useful to communicate the information generated by the model to the participants. Likewise, while the role of facilitator is noted to be important in most participatory activity [46,49], assigning the role of facilitator to one person and the role of chauffeur to another distinctly benefitted the process. Therefore, careful design of the model and tools and the harnessing of the skills of the facilitator and chauffeur are essential for our approach. Another challenge relevant to the approach is the explicit inclusion of qualitative information—in addition to quantitative information—in CuBA. This challenge is recognized as being present in many domains, particularly where scientific or technical information interfaces with human values [40]. Although through our approach the engagement of multiple stakeholders increased the representation of qualitative information in the assessment of cumulative burdens, a qualitative/quantitative tension was found to remain. For instance, this tension became apparent when participants were discussing the perception of residents on noise pollution, the quality of green areas and the traffic situation in a particular street. We noted that our approach could be extended to include the qualitative information as other layers of information in the model, although such qualitative information would have to be elicited and captured beforehand. Perhaps it is relevant to explore the integration of other approaches for capturing qualitative information for stakeholder-based CuBA, such as the one developed by Shrestha et al. [36]. Likewise, in order to fully support stakeholder dialogue in cumulative burden assessment, the approach would need to extend the CuBA model to include health-related indicators/indices. Dealing with the conceptual mismatch related to a cumulative index of environmental burdens is another challenge that needs to be addressed. To do so, it might be necessary to understand interactions among various environmental factors and the influence these may have on each other when those factors occur simultaneously.

In this study, we sought to develop an approach to facilitate science-based stakeholder dialogue while assessing cumulative burdens. Given our intention to construct and test a comprehensive approach and provide initial insights about its applicability, we conducted workshops in Dortmund and Munich with five and seven participants, respectively. To this end, we adopted an exploratory methodology and conducted a micro-analysis of the workshop data. In doing so, we examined the interactions in depth as mediated by our approach and found some outcomes on social learning and co-production of knowledge. Yet, looking at research into science-policy interfaces and other literature on social learning and knowledge co-production, we consider that other factors, for example prior knowledge of the topic and study area by stakeholders, the social and institutional context in which the participants’ experience are embedded, and the group dynamics during the workshop, might have influenced the way participants access and use the models and the tool. Therefore, further research may be worthwhile on how such an approach enables or constrains participants in CuBA in various institutional, social and political contexts. Other methodologies, such as a more controlled experiment with a larger number of participants, would also be worthwhile to derive more quantitative results on various elements of the framework that may be generalized.

## Figures and Tables

**Table 1:** Evaluation of participants’ responses on usefulness of the tool and model specifications.

Tool and Model Specifications	Very Much Agree	Agree	Somewhat Agree	Disagree	Very Much Disagree
The model integrated into the MapTable was useful for identifying ‘hotspots’ relevant to cumulative burden assessment across social vulnerability					
	2	4	2	0	1
The following information provided during workshop was useful for cumulative burden assessment					
Individual indicators (air quality, noise, migration background, etc.)	3	4	2	0	0
Cumulative environmental index	2	1	3	2	1
Social vulnerability index	2	5	1	1	0
Integrated indicator on environment and social vulnerability (only for Dortmund)	0	0	1	2	1
The following information was useful to delineate area for resource allocation					
Information on environmental factors	2	0	7	0	0
Information on social factors	1	6	1	1	0

Note: This evaluation is based on only four participants in Dortmund and five participants in Munich.

**Table 2:** Evidence of communication support provided by our interactive stakeholder-based CuBA approach.

Elements	Activities	Descriptions and Examples of Evidence
Active dialogue among participants with a variety of expertise and profession experience	Openness and freedom to share ideas, opinions	Several subjects were raised for discussion, as well as new ideas and opinions being introduced openly
	Building on meanings proposed by others to produce alternative meanings	Discussions raised on ‘what is more risky, dangerous PM_10_ or noise’. Affirming this, another participant added: ‘PM_10_ is defined stricter in law but noise is subjective […]’. To this argument an alternative perspective was proposed by another participant, who stated: ‘most of the noise is produced by road traffic and most of the fine dust particles too. This means it should be the same point, actually’ (00:05:12–00:06:04 Dortmund)
	Support of the discussion by the facilitator	In both workshops, in several instances the facilitator kept the group focused on the task (observer notes)
Questioning an underlying assumption in the model	Openly agreeing and disagreeing on various aspects of model	40 µg as threshold value for air pollution was acknowledged to be relevant as it is set by law (00:18:41 Dortmund) Disagreement on the use of 70 dbA, which is acknowledged as the remediation value rather than precautionary value, and agreement reached on 55 dbA (00:18:41 Dortmund; 00:02:16–00:03:38 Munich)
	Raising concerns and critical discussions related to the model	Concerns raised on averaging out of noise levels from three sources (industry, tram, street) in cumulative index (00:28:33 Dortmund), on balancing one factor by another in the aggregated index (00:24:26 Dortmund), on absence of indicator on quality of green areas (00:19:11 Munich) Critical discussion on the implication of using Euclidean distance (distance as crow flies) to measure accessibility to green areas (00:07:46–00:09:12 Dortmund)
	Seeking explanation from the facilitator to better understand the model	Facilitator explained the use of absolute vs. relative population data in social vulnerability indicator to emphasize the number of vulnerable populations (00: 38: 24 Munich)
	Providing feedback to improve the model	Acknowledge the need for other data to deliberate on cumulative burdens such as location of hospitals, schools, other social vulnerability indicators, quality of green areas, traffic volumes, health status (open-ended questionnaire)
Exchange of each other’s perspectives	Different viewpoints shared on same topic	One participant explained the benefit of including areas of at least 1 ha as used in the model so that people can experience the natural environment; another stated quality of green areas to be important, with small green areas and also non-green areas being relevant for children (00:12:07–00:14:39 Dortmund); and yet another explained the quality of green areas in general (00:19:09; 00:20:49 Munich)
	Explaining one’s viewpoint in relation to what is visualized in the MapTable	Changing the threshold noise value from 70 dbA to 55 dbA based on own experience (17:54–18:41 Dortmund)
	Explaining one’s viewpoint in relation to what is not yet visualized in the MapTable	Explaining differences in peoples’ subjective perception of noise (00:04:34–00:05:31 Munich)

**Table 3:** Evidence on information support provided by our interactive stakeholder-based CuBA approach.

Elements	Activities	Description and Examples of Evidence
Dynamic exploration of information	Viewing of information at various scales-street, neighbourhood, through to city-wide	One participant remarked that the tool can display values of indicators per street and that it is exciting to see other sub-zones besides Nordstadt, which has always been a broad-funding area (00:31:50 Dortmund) Spatial resolution of environmental indicators could be refined more (observer notes) Tool enabled to get an overview of differences within a neighbourhood (observer notes)
	Seeking information via various combinations of indicators and indices	Assessment of indicators individually or in combination to produce an index and by overlaying one indicator with another (screen capture)
	Changing assumptions in the model and visualizing those changes in real-time	Participants changed threshold of noise level from 70 dBA to 55 dBA to see the difference (screen capture)
	Seeking guidance on using the tool	Participant asked for guidance, such as: ‘could we make it smaller so that we can have an overview again?’ (00:06:19 Munich)
Elicitation and combination of various knowledge types	Drawing on own knowledge to explain or understand the existing information	One participant explained to another the concept of threshold values in planning (00:03:01 Munich)
	Highlighting information not included in the model	Participants remarked that important parks known to them were missing (1:00:38–1:01:50 Dortmund); importance of quality of green parks for the city (00:19:09 Munich)
	Supplementing the information provided by indicators/indices to further contextualize the information	A participant noticed an area that had above-average values for all social vulnerability indicators (SGBII, migrant background, number of kids, older adults). This was further elaborated upon by another participant working in the area stating that the area itself is being considered in the Social City programme (00:52:02–00:52:12 Munich)

## Appendix A

**Table A1:** List of indicator maps used during the workshops.

Dimension	Domain	Description of Indicators
Environmental burdens	Air Quality	Annual average concentration of PM_10_ (µg/m^3^)
		Annual average concentration of NO_2_ (µg/m^3^)
		Number of days PM_10_ exceed the limit of 40 µg/m^3^ (d/a)
	Noise Nuisance	Noise level from individual sources (industries, street and tram) in decibels (dBA)
		Logarithmic aggregation of noise levels (industries, street and tram) in decibels (dBA) (for Munich)
Environmental benefits	Green spaces	Accessibility to green areas >1 ha within walking distance
		Accessibility to forest areas >1 ha within walking distance
Social vulnerability	Sensitive population	Number of children aged 6–11 years (persons/625 m^2^)
		Number of adults aged 65 years and over (persons/625 m^2^)
	Social and economic	Number of people with migration background (persons/625 m^2^)
		Number of people receiving SGB II ^1^ (persons/625 m^2^)
		Number of people receiving SGB XII ^2^ (persons/625 m^2^)

^1,2^ Social welfare recipients: SGB II for working age population receiving assistance, an indicator of unemployment; SGBXII to provide basic security covering old age, disability, living assistance, an indicator of those living below the poverty line.

**Table A2:** Environmental standards used during the workshops.

Environmental Indicators	Threshold Values
Annual average PM_10_ concentration	40 µg/m^3^
Annual average NO_2_ concentration	40 µg/m^3^
Annual average noise level	70 dBA
Distance to green spaces >1 ha	500 m
